# KinasePred: A Computational Tool for Small-Molecule Kinase Target Prediction

**DOI:** 10.3390/ijms26052157

**Published:** 2025-02-27

**Authors:** Miriana Di Stefano, Lisa Piazza, Clarissa Poles, Salvatore Galati, Carlotta Granchi, Antonio Giordano, Luca Campisi, Marco Macchia, Giulio Poli, Tiziano Tuccinardi

**Affiliations:** 1 Department of Pharmacy, University of Pisa, 56124 Pisa, Italy; miriana.distefano@farm.unipi.it (M.D.S.); lisa.piazza@phd.unipi.it (L.P.); carlotta.granchi@unipi.it (C.G.); marco.macchia@unipi.it (M.M.); tiziano.tuccinardi@unipi.it (T.T.); 2 Telethon Institute of Genetics and Medicine, 80078 Naples, Italy; c.poles@tigem.it; 3 Genomics and Experimental Medicine Program, Scuola Superiore Meridionale (SSM, School of Advanced Studies), 80078 Naples, Italy; 4 Sbarro Institute for Cancer Research and Molecular Medicine, Center for Biotechnology, College of Science and Technology, Temple University, Philadelphia, PA 19122, USA; giordano@temple.edu; 5 Flashtox srl, Via Tosco Romagnola 136, 56025 Pontedera, Italy; l.campisi@flashtox.com

**Keywords:** kinase, machine learning, virtual screening

## Abstract

Protein kinases are key regulators of cellular processes and critical therapeutic targets in diseases like cancer, making them a focal point for drug discovery efforts. In this context, we developed KinasePred, a robust computational workflow that combines machine learning and explainable artificial intelligence to predict the kinase activity of small molecules while providing detailed insights into the structural features driving ligand-target interactions. Our kinase-family predictive tool demonstrated significant performance, validated through virtual screening, where it successfully identified six kinase inhibitors. Target-focused operational models were subsequently developed to refine target-specific predictions, enabling the identification of molecular determinants of kinase selectivity. This integrated framework not only accelerates the screening and identification of kinase-targeting compounds but also supports broader applications in target identification, polypharmacology studies, and off-target effect analysis, providing a versatile tool for streamlining the drug discovery process.

## 1. Introduction

Protein kinases constitute a diverse family of enzymes that regulate numerous cellular processes through phosphorylation, a key post-translational modification that modulates protein function [1]. By transferring phosphate groups to serine, threonine, or tyrosine residues on substrate proteins, kinases control critical cellular activities, including signal transduction, cell proliferation, differentiation, metabolism, and apoptosis [2]. These enzymes function as molecular switches, finely tuning cellular responses to external and internal signals, thereby maintaining cellular homeostasis and enabling proper physiological functions [3]. Given their fundamental role in cell signaling, dysregulation of kinase activity is implicated in a wide range of diseases, especially cancer, where aberrant phosphorylation can drive uncontrolled cell growth and tumor progression [4]. In many cancers, mutations, amplifications, or translocations in kinase genes lead to constitutive activation of kinase signaling pathways, ultimately resulting in the evasion of growth control mechanisms and the promotion of malignancy. Consequently, kinases have emerged as important therapeutic targets, particularly in oncology, where several kinase inhibitors, such as imatinib and erlotinib, have been approved for clinical use. These drugs have significantly advanced cancer treatment by selectively targeting kinases involved in tumorigenesis and other pathological processes [5]. The clinical success of these inhibitors has not only provided proof-of-concept for targeting kinases but also inspired the development of numerous next-generation inhibitors to overcome resistance and improve patient outcomes. Despite the clinical success of many therapeutic agents, the unintended inhibition of kinases as off-targets remains a significant challenge in drug development [6]. Kinase signaling networks are inherently complex, with overlapping pathways and multiple isoforms that contribute to redundancy and resistance mechanisms. While many drugs are specifically designed to inhibit non-kinase targets, their interaction with kinases—due to the high structural conservation of the ATP-binding pocket and other binding domains across kinase families—can lead to off-target effects. Such unintended kinase inhibition can disrupt essential physiological processes, resulting in adverse effects, including cardiotoxicity, hepatotoxicity, or immune dysregulation. For example, the off-target inhibition of kinases like Janus kinases (JNKs) or p38 mitogen-activated protein kinases (MAPKs) has been associated with metabolic and inflammatory disturbances, highlighting the broader implications of these interactions [7]. Traditional experimental approaches, such as mass spectrometry-based phosphoproteomics, have been widely used to map kinase–substrate interactions and provide invaluable insights into cellular signaling [8]. However, these methods are resource-intensive, costly, and often limited in scalability, posing challenges for their application to large-scale studies or comprehensive mapping of kinase–substrate networks in complex biological systems. Despite advances in improving the throughput of mass spectrometry, many phosphorylation sites remain unidentified, and their upstream kinases are yet to be mapped. This incomplete knowledge, combined with the widespread involvement of kinases in cellular signaling and their structural conservation, increases the risk of unintended interactions with therapeutic molecules, even those designed for non-kinase targets. A deeper understanding of kinase–substrate relationships and kinase-mediated signaling is critical to identify and mitigate off-target effects, ultimately improving the safety and selectivity of therapeutic agents.

To address these challenges, computational approaches, particularly those leveraging machine learning (ML), have gained prominence. ML-based methods have demonstrated remarkable accuracy and scalability in predicting protein-specific interactions, integrating diverse data sources to uncover complex relationships [9,10,11]. The use of ML has revolutionized the identification of drug leads through virtual screening (VS), enabling the discovery of novel kinase inhibitors and validating the reliability of these predictive protocols. Additionally, ML approaches provide significant advantages in terms of efficiency, allowing rapid analysis of large datasets and uncovering subtle correlations that traditional methods may overlook. For instance, deep learning frameworks have been employed to predict kinase–ligand binding affinities using large-scale datasets of kinase–inhibitor interactions. Tools like DeepDTA and AtomNet apply neural network architectures to learn the intricate features of molecular binding, offering highly accurate predictions [12]. Another notable application of ML is the use of reinforcement learning to design kinase inhibitors with improved selectivity. These algorithms iteratively optimize molecular structures based on reward functions that incorporate kinase specificity and drug-likeness metrics [13]. In addition to predictive modeling and optimization, convolutional neural networks (CNNs) have been applied to structure-based drug discovery. For example, the application of CNNs in 3D molecular representation has shown promise in identifying kinase inhibitors by analyzing the spatial configuration of kinase active sites and potential ligands. This approach, implemented in tools like DeepChem and similar platforms, has proven effective in recognizing subtle structural features important for binding specificity [14]. Within the field of ML-driven drug discovery, explainable artificial intelligence (XAI) represents a revolutionary approach by addressing one of the most critical challenges in applying ML: the interpretability of predictive operational models. While traditional AI and ML algorithms, such as deep neural networks, are highly effective in processing complex datasets, their “black box” nature often limits their application in domains like drug discovery, where transparency and understanding are essential. In drug discovery, XAI enables researchers to interpret the rationale behind ML-based predictions, such as identifying key molecular features that contribute to a compound’s activity or off-target effects. This interpretability not only builds trust in AI-driven predictions but also accelerates hypothesis generation by providing actionable insights into molecular interactions, toxicity profiles, or binding affinities. For example, XAI techniques like SHAP (SHapley Additive exPlanations) and LIME (Local Interpretable Model-agnostic Explanations) [15] allow researchers to understand the contributions of individual features, such as chemical substructures, to a compound’s predicted efficacy. Such insights facilitate the design of safer and more effective drugs by highlighting modifiable regions within molecules [16].

In this context, we present KinasePred, a computational platform designed to predict the potential inhibitory activity of small molecules against kinase targets. By integrating data from the ChEMBL database [17], KinasePred offers a robust framework for identifying potential kinase targets across diverse families. Furthermore, the application of a robust XAI approach was used to detect rational patterns able to logically explain the predicted bioactivity, providing information about the binding mode of small molecules. The platform has been validated through the experimental screening of small molecules predicted to exhibit kinase activity. Compounds identified as potentially active were subsequently tested on a panel of 20 kinases, revealing significant activity and demonstrating the predictive power of the protocol. Building on these results, target-specific predictive protocols were developed for individual kinase targets, providing a more precise approach to predict activity on specific kinases. The overall workflow of our study, including the step-by-step procedures followed, is illustrated in Supplementary Figure S1. By accurately predicting kinase interactions, KinasePred not only facilitates the design of selective kinase inhibitors but also supports drug-repurposing initiatives and helps identify off-target interactions that may lead to adverse effects. Such platforms are invaluable for addressing the complexities of kinase signaling in diseases such as cancer, neurodegeneration, and autoimmune disorders, where kinase dysregulation is a primary driver. Ultimately, KinasePred exemplifies the potential of computational tools to accelerate drug discovery, improve therapeutic outcomes, and deepen our understanding of kinase-mediated signaling in health and disease.

## 2. Results and Discussion

### 2.1. Generation and Evaluation of ML Operational Models

As a first step to develop reliable ML operational models (hereinafter referred to as “models”, for simplicity) for predicting the activity of small molecules on kinases, we generated a curated dataset using data from high-quality literature sources, and the data were systematically processed to ensure robustness and accuracy. The focus was placed on the kinase family reported in the first ring of the ChEMBL Protein Target Tree version 29, resulting in the selection of 440 kinase targets as single proteins for Homo sapiens organisms. The dataset we generated sourcing from ChEMBL29 included only kinase-targeting compounds with reliable activity data (IC_50_, *K*_I_, *K*_d_, or EC_50_ ≤ 10 μM) and a high confidence score for target assignment. After removing duplicates, inconsistencies, and chemically invalid structures, we eventually obtained a curated comprehensive dataset of ligands targeting human protein kinases, which constituted the active compounds to be employed for developing ML models. These compounds were balanced by inactive decoys retrieved from the ZINC15 database (see Materials and Methods for details). These decoy molecules were processed using the same pipeline as for the active compounds to ensure consistent chemical representations. The final dataset, which included the compounds retrieved from ChEMBL29 and ZINC15, was then split into training and test sets (including 80% and 20%, respectively, of the initial data). Using the selected training data, we developed nine supervised ML classification models aimed at predicting the potential activity of small molecules against kinase targets. This was achieved by combining three different algorithms with three distinct molecular representation methods. In particular, Random Forest (RF), Gaussian Naïve Bayes (GNB), and Multi-Layer Perceptron (MLP) algorithms were used in combination with Morgan, RDKit, and PubChem Fingerprints (FPs), as detailed in the Materials and Methods section. Each model was subjected to an optimization process aimed at identifying the best hyperparameter configuration for maximizing its performance. A 10-fold cross-validation (CV) analysis was then performed for each optimized model to obtain an overview of its predictive capacity (see Materials and Methods for details). The performance of all models was evaluated and ranked according to the values of Matthews Correlation Coefficient (MCC) obtained through CV analysis (Supplementary Table S1), since it provides a balanced measure of model performance. The MLP–Morgan model achieved the highest MCC value (0.96 ± 0.01), outperforming all other models. This model also excelled across all complementary metrics used for the statistical evaluation, demonstrating exceptional reliability and robustness. Precisely, it achieved a Balanced Accuracy (BA) of 0.98 ± 0.00, a Negative Predictive Value (NPV) of 0.98 ± 0.00, a Precision of 0.97 ± 0.01, a Recall of 0.98 ± 0.01, and a Specificity of 0.97 ± 0.01. These results highlight that the MLP–Morgan model was not only highly accurate but also robust for and well-suited to practical predictive tasks in kinase activity predictions, offering a reliable foundation for decision-making in drug discovery workflows. In contrast, the worst performing model was obtained by the combination of GNB algorithm with PubChem FPs, which yielded an MCC of 0.55 ± 0.02. This poor performance extended across all complementary metrics (Supplementary Table S1). 

Subsequently, the performance of each model was further assessed using the test set to rigorously assess the generalization ability of the models on unseen data. This step was critical for evaluating their realistic predictive potential, as it provided insights into their performance when applied to novel compounds. The results obtained during test set validation confirmed the trends observed in the CV phase (Table 1).

The MLP–Morgan model consistently demonstrated the highest performance across all evaluated metrics, with an MCC of 0.96, Recall of 0.99, and Specificity of 0.98, translating to an excellent BA of 0.98. These results underscored its ability to generalize well to novel compounds, maintaining a high degree of Precision and Reliability in distinguishing active from inactive compounds. The robust performance of the MLP–Morgan model across key metrics such as NPV (0.99) and Precision (0.99) further highlighted its potential as a reliable predictive tool for kinase activity. The other MLP-based models, based on PubChem and RDKit FPs, also demonstrated strong performance, with MCC values of 0.94 and 0.93, respectively. These models maintained good Recall, Specificity, and BA values, reinforcing the versatility of the MLP algorithm across different molecular representations. Among models based on different algorithms, the RF–Morgan model performed competitively, achieving an MCC of 0.93 and a BA of 0.96, suggesting that RF, when paired with Morgan FPs, can also serve as an effective approach for predicting kinase activity. In contrast, models based on GNB algorithm consistently exhibited lower performance. In particular, the GNB–PubChem model achieved an MCC of only 0.53 and a Specificity of 0.54, indicating significant limitations in identifying inactive compounds. Even the best-performing GNB model (GNB–Morgan) showed an MCC of 0.78, a result substantially lower than those of showed by the MLP-based and RF-based models.

These findings underscored the challenges associated with using GNB algorithm for complex tasks such as kinase activity prediction, and they highlighted its sensitivity to the choice of molecular representation. Overall, these results confirmed the critical importance of selecting the appropriate combination of algorithm and molecular FPs for optimizing the predictive performance of the models. The superior performance of the MLP–Morgan model demonstrated the strength of this synergy, emphasizing the utility of Morgan FPs in capturing structural features critical for kinase activity predictions.

To further validate the performance of the MLP–Morgan model, an external validation was conducted using an independent dataset derived from ChEMBL30. This dataset was constructed by using kinase data from ChEMBL30 not present in ChEMBL29. These data were then processed identically to those gathered from ChEMBL29 and combined with an equal number of randomly retrieved decoys sourced from the ZINC15 database (see Materials and Methods for details). By evaluating the model on an independent dataset, we aimed to thoroughly test its generalization ability and confirm its high reliability. The results summarized in Table 2 demonstrated the strong predictive performance of the MLP–Morgan model, which achieved a high MCC of 0.88, reflecting the strong reliability of its predictions of both active and inactive compounds. This was further highlighted by the very high BA value of 0.94. Precision was particularly noteworthy at 0.97, underscoring the ability of the model to accurately predict active compounds while minimizing false positives. Moreover, Specificity reached a value of 0.98, indicating the model’s robustness in identifying inactive compounds, even in the presence of challenging decoys. These metrics collectively confirmed the MLP–Morgan model’s ability to effectively classify unseen data, suggesting its utility as a predictive tool for kinase activity in diverse applications.

### 2.2. Explainability of the Best Model

**Figure 1:**
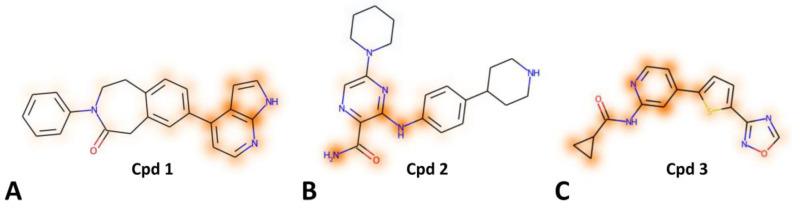
Results obtained from SHAP analysis for the ROCK2 inhibitor **1** (**A**), the BTK inhibitor **2** (**B**), and the GSK-3β inhibitor **3** (**C**) recently co-crystallized with their corresponding kinase target. The orange-colored moieties indicate a greater impact on the prediction.

To assess the interpretability of the ML predictions, we conducted an explainability analysis using the SHAP method to identify the structural moieties of the molecules that most significantly contributed to their predicted activity (see Materials and Methods for details). For this purpose, three representative kinase ligands recently co-crystallized with their corresponding well-known target, not included in the ChEMBL29 dataset used for the training and validation of our kinase-family ML model, were randomly selected (see Materials and Methods for details): a 8-(azaindolyl)-benzoazepinone inhibitor (**1** in Figure 1A) of Rho-Associated Coiled-Coil Containing Protein Kinase 2 (ROCK2), 5-(piperidin-1-yl)-3-{[4-(piperidin-4-yl)phenyl]amino}pyrazine-2-carboxamide inhibitor (**2** in Figure 1B) of Bruton’s tyrosine kinase (BTK), and N-(4-(5-(1,2,4-oxadiazol-3-yl)thiophen-2-yl)pyridin-2-yl)cyclopropanecarboxamide inhibitor (**3** in Figure 1C) of Glycogen Synthase Kinase 3 Beta (GSK-3β). The three molecules were correctly predicted as active against kinase targets by our kinase-family predictive model. The SHAP analysis was thus applied to compounds **1-3** to pinpoint the structural features driving their predicted activity, and the results were compared to the corresponding ligand–protein X-ray structures available for these compounds (PDB codes 9EP8 [18], 8GC7 [19], and 8QJI [20], respectively) to ensure consistency between the SHAP results and their experimentally determined binding modes. Notably, the SHAP evaluation was able to identify the portion of each compound interacting with the hinge region of the corresponding target and that was, thus, responsible for anchoring the inhibitors into the kinase binding site, assuring a stable ligand–protein interaction.

In particular, the 8-azaindole moiety of compound **1**, highlighted by the SHAP analysis with intense orange shading (Figure 1A), was identified as the principal contributor to its prediction as kinase inhibitor. In fact, such bicyclic portion is responsible for the formation of two H-bonds with residues belonging to the hinge region of ROCK2 kinase (E170 and M172) [18]. The SHAP analysis performed on compound **2** highlighted the carboxamide moiety and the adjacent amino group of the ligand as being mainly responsible for the activity prediction of this compound. Notably, the ligand carboxamide group anchors the inhibitor to the hinge region of BTK, forming H-bonds with M477 and E475. Moreover, the amide NH2 group forms an additional water-mediated interaction with T474 [19]. Interestingly, although the amino group connecting the two aryl rings of the ligand is not involved in interactions with the hinge region of BTK, it appears to form an intramolecular H-bond with the carboxamide oxygen of the compound, thus representing a key part of the central hinge-binding scaffold of the ligand. Finally, the SHAP analysis properly recognized the pyridinylcyclopropanecarboxamide moiety of compound **3**, intensely highlighted in orange in Figure 1C, as the hinge-binding portion of the ligand, which allows its interaction with the hinge region of GSK-3β kinase. In particular, the compound forms two H-bonds with residue V135 thanks to its pyridine nitrogen and its amide group [20]. Overall, the results shown herein demonstrate that the SHAP analysis is able to identify the structural moieties that most impact on the interaction of the ligands with their corresponding target. This agreement between SHAP results and experimental evidence validates the interpretability of our kinase-family predictive model and demonstrates the reliability of SHAP in identifying chemically relevant features in predictive analyses.

### 2.3. Virtual Screening and Experimental Evaluation

**Figure 2:**
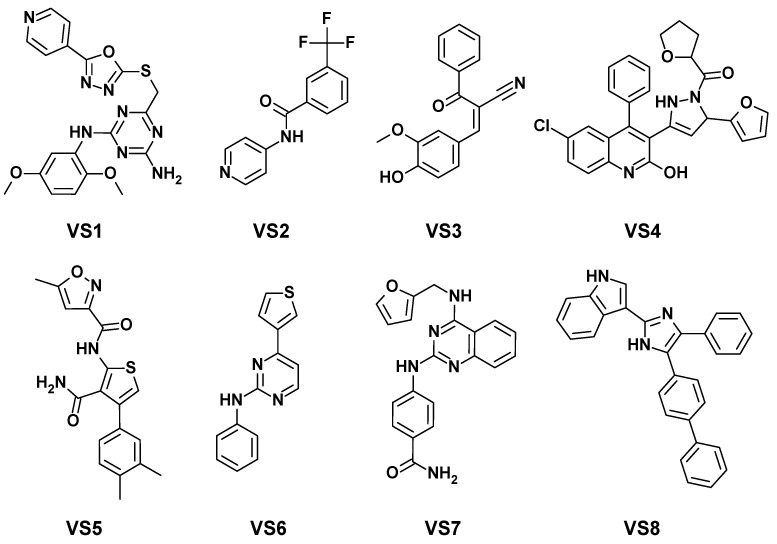
Chemical structures of the eight molecules identified through virtual screening.

A VS study was carried out using the MLP–Morgan model, identified as the best-performing predictive tool for kinase-family activity. The main goal of this screening was to select compounds for experimental testing, serving as a critical step to validate the predictive performance of the model while simultaneously identifying new potential kinase inhibitors. The compounds used for the VS study were derived from a meticulously refined dataset of compounds present in ChEMBL31. Initially, all kinase-associated compounds from the full ChEMBL31 database were removed to isolate only kinase-independent entries, i.e., compounds tested for bioactivity toward targets other than kinases. A further analysis was performed to compare the remaining dataset against ChEMBL29, thus ensuring no overlap with it. The resulting dataset, comprising 732′749 unique compounds, underwent rigorous preprocessing to ensure the use of high-quality data, consistent with the previously generated datasets. Using the MLP–Morgan model, 36′302 compounds (approximately 5% of the dataset) were identified as potential kinase inhibitors. The ChemBridge database was inspected to check for the potential commercial availability of the selected compounds, which resulted in the identification of a set of 61 ready-to-buy compounds in suitable amounts. To refine this selection, the compounds were subjected to structural clustering, which produced eight clusters of structurally similar molecules, and a representative of each cluster (compounds **VS1-8** in Figure 2) was then selected after visual inspection.

The eight molecules were tested against a rationally, carefully selected panel of kinases. The selection began by identifying the 40 kinase targets with the highest number of associated compound bioactivities reported in the literature, a metric reflecting their key biological and pharmacological significance. These targets were considered highly relevant for kinase research and drug discovery. To ensure diversity, the sequences of these kinase targets were retrieved in FASTA format from ChEMBL and analyzed using ClustalW [21], a sequence alignment tool. The alignment facilitated a clustering analysis, grouping kinases based on sequence similarity. From these clusters, 20 representative kinases (Supplementary Table S2) were selected to provide a diverse and balanced panel that adequately reflected the broader kinase landscape. The experimental evaluation of the eight selected compounds against the kinase panel revealed that six out of eight compounds demonstrated inhibitory activity. Specifically, compounds **VS2–VS7** exhibited inhibition greater than 50% against at least one kinase target (Table 3).

As shown in Table 3, **VS7** emerged as the most active compound, completely abolishing LRKK2 catalytic activity (100% inhibition); strongly inhibiting NTRK1 (90%), SYK (90%), CSF1R (89%), and IRAK4 (80%); and showing considerable activity also against PTK2 (66%) and LCK (53%). Notably, **VS7** previously demonstrated potential antimalarial activity [22]. The findings herein presented expand the knowledge about the biological activity of **VS7**, highlighting its significant inhibitory effects on multiple kinase targets and suggesting its potential use for broader therapeutic applications. The complete inhibition of LRKK2 highlights its promise for neurodegenerative diseases [23], while its strong inhibition of CSF1R and NTRK1 [24,25] underscores its potential relevance in oncology and nervous system disorders. The combination of broad and potent activity makes **VS7** a compelling candidate for further preclinical evaluation. Compound **VS6**, previously referenced in the literature for its structural relevance in developing inhibitors targeting histamine release from basophils [26], demonstrated broad activity as well, with strong inhibition of CSF1R (89%), and considerable activity against MAPK8 (63%), LRKK2 (60%), and SYK (57%). This profile suggests **VS6** as a potential multi-target inhibitor with therapeutic relevance in areas such as oncology and immune regulation [27,28]. The simultaneous inhibition of CSF1R and SYK is particularly notable, as it may enhance its effectiveness in immuno-oncology or inflammatory conditions. Compounds **VS2** and **VS4**, which were previously investigated for their potential antimalarial activity [29,30], both showed inhibition of two different kinase targets of the panel. In particular, **VS4** demonstrated a strong inhibition of CSF1R (90%), together with a remarkable activity against BRAF (56%), highlighting its potential application for targeting oncogenic pathways. In fact, the strong activity against CSF1R, a key regulator of macrophage function, and its selectivity over BRAF suggest possible applications in immuno-oncology. Differently, compound **VS2** showed good inhibitory activity against ROCK2 kinase (74%) and a lower but still considerable inhibition of LRKK2 (50%), suggesting its potential as a selective inhibitor of ROCK2 kinase. Finally, compounds **VS3** and **VS5** were found to inhibit with interesting activity (54%) a single target among the panel of tested kinases, corresponding to LRKK2 and CSF1R, respectively. Notably, both compounds were previously studied, with **VS3** investigated across a range of cancer-related targets and **VS5** examined for its potential antimalarial activity [31]. The experimental results herein obtained collectively highlight the reliability of our kinase-family predictive model, since six out of the eight representative compounds tested (**VS2–VS7**) showed considerable inhibitory activity against at least a single kinase target belonging to the panel employed for biological evaluations. Moreover, half of the tested compounds (**VS2**, **VS4**, **VS6**, and **VS7**) showed activity against more than one target and very interesting inhibitory potencies, reaching a percentage of inhibition up to 74–100%.

### 2.4. Generation and Evaluation of Target-Specific Models

In kinase-focused drug discovery, accurately predicting the specific kinase targets of a compound is critical. While family-level predictions provide a general understanding, they fail to capture the detailed binding profiles of compounds. This can hinder the identification of selective inhibitors or compounds with a polypharmacological profile, both of which are vital for developing effective and safe therapeutics. Thus, target-level predictions provide a more specific and actionable insight, enabling the prioritization of compounds for experimental validation. To address this challenge, 230 different RF classifiers were developed to predict the activity of small molecules against the most representative kinase targets among those considered for generating the kinase-family predictive model (see Materials and Methods for details). The choice of this algorithm rather than MLP was driven by the ability of RF to produce reliable models even from small datasets. The performance of each generated model was evaluated through a 5-fold CV scheme, where for each fold, the dataset of each model was randomly split into training (70%) and testing (30%) subsets, and employing the same metrics used to test the reliability of the kinase-family models. Table 4 reports the average statistical results obtained by the whole ensemble of RF models, which showed MCC and BA values of 0.49 and 0.83, respectively, indicating a significant overall predictive performance. Additionally, Precision (0.89) and Recall (0.89) were strong, meaning that the RF models were overall effective at both correctly predicting active compounds and minimizing false positives. However, the models struggled with inactive compounds, as reflected by their relatively low NPV (0.55) and Specificity (0.66). This suggested that, while RF models performed robustly for actives, they often misclassified inactive compounds as active. However, this limitation was likely driven by the imbalanced nature of the datasets used to develop the target-specific RF models. In fact, the number of active compounds in each target-specific dataset was generally higher than the number of inactive molecules.

Imbalanced datasets, where one class significantly outweighs the other, pose a major challenge in predictive modeling. Models trained on such datasets tend to favor the majority class, leading to biased predictions. When the majority class is constituted by active compounds, as in our case, this bias could result in high Precision and Recall (due to the correct prediction of active compounds), but poor NPV and/or Specificity (due to the misclassification of inactive compounds). This imbalance complicates the development of models whose goal is the accurate prediction of both active and inactive compounds, as it skews model training toward the prediction of actives. To evaluate how this class imbalance impacted the performance of the RF models, the “inactive ratio”, defined as the ratio between inactive and active compounds in a dataset, was calculated for each of the 230 target-specific datasets used for developing the corresponding RF models. Datasets with an inactive ratio < 25% were considered to be highly imbalanced, and the corresponding RF models were removed from the overall performance analysis. The overall performance of the reduced ensemble of RF models, which included 130 models related to the 130 kinase targets with sufficiently balanced datasets, was then re-evaluated. As reported in Table 4, the removal of models with highly unbalanced datasets generated an improvement in the average MCC, which increased from 0.49 to 0.59. This indicated a better overall balance in the models’ predictions, showing higher performance in distinguishing between active and inactive compounds. This improvement was also underscored by a significant increase in NPV from 0.55 to 0.74, indicating enhanced ability to correctly identify inactive compounds. Additionally, Specificity increased from 0.66 to 0.78, suggesting improved performance in avoiding false positives. As expected, we also observed a slight decrease in Recall (from 0.89 to 0.85) and especially in Precision (from 0.89 to 0.80), highlighting more difficulties in properly identifying active compounds. The improvements in MCC, NPV, and Specificity highlighted the importance of working with more balanced datasets to mitigate the noise introduced by highly skewed predictions. Despite the slight decreases in Precision and Recall, these trade-offs were acceptable given the overall gains in the reliability of the models and in their ability to correctly predict inactive compounds. The use of the updated set of RF models represented a more robust and balanced approach for target-specific kinase activity predictions. These results demonstrated the value of filtering out targets with highly unbalanced datasets to improve the overall reliability of predictions.

### 2.5. Experimental Validation

**Figure 3:**
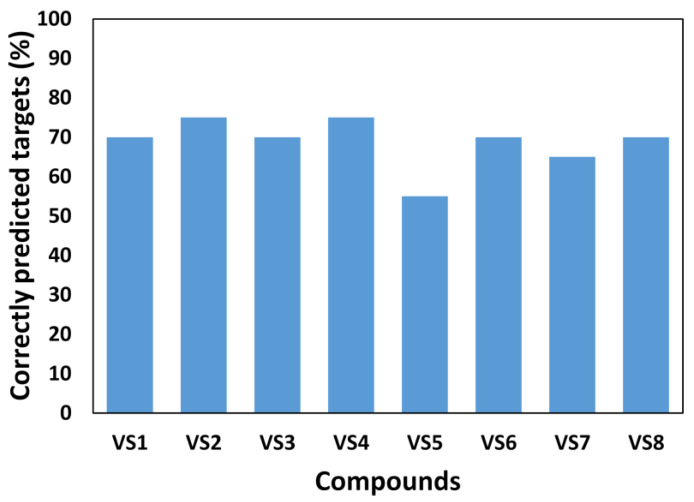
Success rates of single-target RF models in predicting kinase inhibitory activity for the 8 selected compounds (**VS1–8**). The success rate represents the percentage of correctly predicted kinase activities obtained for each compound against the 20 experimentally tested targets.

To assess the performance of the target-specific RF models, the predictions of the eight tested compounds (**VS1–8**) were compared to the experimental results obtained from biological assays. In practice, each compound was evaluated by the 20 single-target RF models related to the 20 kinase targets considered for the experimental evaluation. On the basis of the experimental results, the percentage of correct predictions out of all 20 predictions obtained for each compound was then calculated, and the results are displayed in Figure 3.

Overall, the predictive performance of the single-target RF models varied across compounds, with success rates ranging from 55% to 75%. Compounds **VS2** and **VS4** achieved the highest performance, with correct activity predictions for 15 out of 20 targets, corresponding to a success rate of 75%. A similarly high performance was also observed for compounds **VS1**, **VS3**, **VS6**, and **VS8**, for which a success rate of 70% (corresponding to 14 out of 20 targets correctly predicted) was obtained. Lower success rates, corresponding to 55% and 65%, were observed for **VS5** and **VS7**, respectively, since the single-target RF models were able to correctly predict their activity against 11 and 13 targets, respectively, out of the 20 kinases included in the panel. Nevertheless, our models showed remarkable overall reliability, showing a success rate of at least 70% for six out of the eight tested compounds (**VS1-4**, **VS6**, and **VS8**), and proving better than random choice for the remaining two compounds (**VS5** and **VS7**). These results highlight the reliability of RF models for target-specific activity predictions, particularly when sufficient training data are available. While lower success rates were observed for a few compounds, these discrepancies can be explained by data imbalance and compound-specific characteristics. Overall, the results demonstrate that the single-target RF models provide accurate and robust predictions, supporting their utility in kinase target identification tasks.

## 3. Materials and Methods

### 3.1. Modeling Datasets

Our primary data sources were ChEMBL29 and ChEMBL30 (last updated in July 2021 and February 2022, respectively) for positive instances [17], and ZINC15 for negative instances [32]. Data retrieved from ChEMBL29 were used to train and internally test the ML models, while data retrieved from ChEMBL30, after removing those shared with ChEMBL29, were used for external evaluation. In particular, we focused on the kinase family reported in the first ring of the ChEMBL Protein Target Tree, selecting 440 kinase targets from Homo sapiens to create a comprehensive dataset of human protein kinases. Only compounds with activity records related to the selected kinase targets and corresponding to IC_50_, *K*_i_, *K*_d_, or EC_50_ values ≤ 10 μM, with a confidence score of at least 9 (indicating a high reliability of target assignment), were retrieved. This high-confidence data ensured the robustness of our training and validation datasets. Data preprocessing involved filtering out compounds containing elements other than H, B, C, N, O, F, Si, P, S, Cl, Se, Br, and I, as well as compounds with valence errors. Compounds with a molecular weight greater than 800 Da or with less than 15 heavy atoms, as well as duplicates, were also removed using MolBook UNIPI [33]. The SMILES (Simplified Molecular-Input Line-Entry System) strings of the remaining compounds were cleaned and standardized using the “washing protocol” from OpenEye, followed by re-ionization using the FixpKa function to standardize ionization states [34]. Data retrieved from both ChEMBL29 and ChEMBL30 underwent the same processing steps to ensure consistency. The compounds obtained from ChEMBL29 were labeled as active and represented the positive instances for model development and validation. To create balanced datasets, an equal number of drug-like decoy molecules was randomly retrieved from ZINC15. These decoy molecules were processed in the same way as the positive instances and labeled as inactive. The final dataset, which contained a total of 118′816 molecules, was then split into a training and test set in an 80:20 ratio. Therefore, the final training and test sets included a total of 95′052 and 23′764 compounds, respectively (50% from ChEMBL29 and 50% from ZINC15). Following the same strategy, the compounds obtained from ChEMBL30 and not present in ChEMBL29 (labeled as active) were combined with an equal number of drug-like decoys retrieved from ZINC15 (labeled as inactive) to create an external test set, which included a total of 7′536 compounds.

### 3.2. Representation of Molecules

The SMILES strings of the selected dataset compounds downloaded from ChEMBL were used to compute three different types of molecular FPs to provide input data for the ML algorithms: Morgan, RDKit, and PubChem FPs. Morgan FPs, also known as circular FPs, are based on Morgan’s algorithm. They represent the structure of the compounds by computing the surrounding environment of each atom, including atomic bonds within a specific distance or radius. The identifiers for each atom are then folded into a fixed-length vector using hashing functions provided by RDKit [35]. In this study, the atomic radius was set to two, and the vector length was fixed at 2048 bits. RDKit FPs are RDKit-specific FPs that identify all subgraphs in the molecules within a particular range of atom sizes. For each identified subgraph, a raw bit ID is computed, and the corresponding bit is set in the FP vector through hashing. Atom types are defined by atomic numbers and aromaticity, while bond types are defined by atom types and bond orders. The FP vector length for RDKit FPs was also set to 2048 bits for consistency with Morgan FPs. PubChem FPs are substructure-based FPs represented by an 881-bit vector calculated using the PyBioMed Python module. These FPs are used to capture the presence or absence of specific chemical substructures within the molecule.

### 3.3. Machine Learning Algorithms

In this study, three different classification algorithms were employed to develop predictive models: RF, GNB, and MLP. For the RF model, the primary hyperparameters optimized were “n_estimators” and “max_features”. The numbers of estimators considered were 100 and 500. For “max_features”, the following options were evaluated: (a) “sqrt”, representing the square root of the total number of features at each node; (b) “None”, indicating that all features were considered; and (c) “log2”, representing the binary logarithm of the total features. The GNB classifier is a probabilistic model based on Bayes’ theorem, assuming independence among predictors. It models the distribution of input features for each class using a Gaussian distribution, estimating parameters such as mean and variance for each feature. Key hyperparameters include “var_smoothing”, which improves numerical stability by adding a portion of the largest variance to feature variances. Both RF and GNB models were implemented with scikit-learn Python library [36].

For the MLP model, deep neural networks were built and trained using the Tensorflow library. The key hyperparameter optimized was the size of the hidden layers, which included the number of neurons, as well as the number of hidden layers. We exhaustively assessed all possible combinations, including architectures with 100, 200, and 1000 neurons per layer.

### 3.4. Performance Metrics

To assess the performance of the ML models developed for kinase target prediction, a comprehensive set of evaluation metrics was employed. These metrics were selected to provide insights into different aspects of the models’ performance, particularly in the context of imbalanced datasets commonly encountered in kinase target prediction tasks. These metrics are based on a confusion matrix derived from a binary classification, which is formed by true positives (TPs), false positives (FPs), true negatives (TNs), and false negatives (FNs). TPs refer to instances that the model correctly identifies as active. FPs occur when the model incorrectly predicts inactive compounds as active. TNs are cases where the model correctly classifies compounds as inactive. FNs occur when the model fails to identify active compounds and classifies them as inactive. The metrics are detailed below:

- Precision

Precision measures the proportion of true positive predictions among all compounds predicted as active:

(1) $$Precision=\frac{TP}{TP+FP}$$

This metric evaluates the model’s ability to minimize false positives, which is important when predicting active compounds.

- Recall

Recall, or Sensitivity, measures the proportion of true active compounds correctly identified:

(2) $$Recall=\frac{TP\;}{TP+FN}$$

This metric is critical for ensuring that active compounds are not overlooked, especially in a target fishing context.

- Negative Predictive Value (NPV)

NPV evaluates the proportion of true negative predictions among all compounds predicted as inactive:

(3) $$NPV=\frac{TN}{FN+TN}$$

This metric is particularly relevant for identifying inactive compounds, ensuring the model avoids false negatives.

- Specificity

Specificity measures the proportion of inactive compounds correctly identified:

(4) $$Specificity=\frac{TN}{TN+FP}$$

This metric complements Recall by focusing on the correct identification of inactive compounds, which is essential for balancing predictions in imbalanced datasets.

- Balanced Accuracy (BA)

BA was used to evaluate the model’s ability to correctly classify both active and inactive compounds while accounting for class imbalance. It is calculated as the average of Recall and Specificity:

(5) $$BA=\frac{Recall+Specificity}{2}$$

This metric ensures that the performance on both classes is equally weighted, avoiding the bias toward the majority class.

- Matthews Correlation Coefficient (MCC)

The MCC measures the quality of binary classifications, taking into account true and false positives and negatives. It provides a balanced measure even in the presence of class imbalance:

(6) $$MCC=\frac{\left(TP\times TN\right)-(FP\times FN)}{\sqrt{\left(TP+FP\right)\times \left(TP+FN\right)\times \left(TN+FP\right)\times (TN+FN)}}$$

An MCC value ranges from −1 to 1, where 1 indicates perfect predictions, 0 indicates no correlation between predictions and true labels, and −1 indicates completely incorrect predictions.

### 3.5. Generation and Evaluation of Models

We combined three different FPs with three distinct ML algorithms to create our models, yielding a total of 9 combinations. We applied an optimization process utilizing Grid Search CV, as implemented in scikit-learn, to determine the optimal hyperparameters settings for all the generated models. Grid Search is a general approach offered by scikit-learn to search for hyperparameters that maximize model performance. It employs a 3-fold stratified CV to explore all feasible hyperparameters combinations, assigning a score to each one. In this study, we employed MCC as the scoring metric of the Grid Search CV. After fine-tuning the hyperparameters, we conducted a further 10-fold CV for each model. We adopted a random training-test set splitting strategy, where 30% of the initial dataset was allocated as the test set for each fold. In order to thoroughly check the performance of the top-scored models, which were selected based on their MCC value, all statistical parameters were taken into account: MCC, Precision, Recall, NPV, Specificity, and BA.

### 3.6. Virtual Screening Dataset

For the VS study, the ChEMBL31 database was utilized as the primary data source. All kinase-related data were removed, and the remaining dataset underwent rigorous processing, identical to the steps applied to data belonging to ChEMBL29 and ChEMBL30. This included removing duplicates, correcting molecular structures; neutralizing charges; and applying filters for molecular properties, such as a minimum of 15 heavy atoms and a molecular weight below 800 Da. After processing, a refined collection of 1′933′197 compounds unrelated to kinases was obtained. To ensure no overlap with ChEMBL29, a comparison of the two databases was performed. The resulting dataset, comprising 732′749 compounds, was used to develop a kinase-independent screening library.

### 3.7. Feature Contribution

In this study, SHAP values were computed to evaluate the contributions of molecular features to the model’s predictions. Originally developed to assess the influence of an individual player on a team’s performance, SHAP values are now used to identify key features that correlate directly with model outcomes. For the binary classifiers used in this study, feature importance was indicated by a sign showing the direction of the contribution: a positive value signifies a contribution to predicting activity. Given the model-specific nature of the SHAP approach, we opted for the Kernel SHAP model-agnostic method provided by the SHAP Python library. Kernel SHAP is based on an extension of LIME, a local approximation method for explaining model decisions [15]. This choice was made due to the substantial computational cost of precisely computing all Shapley values. As a result, a local approximation approach served as a practical alternative for obtaining reliable explanations of ML models [37]. Feature contribution analysis was not performed during CV but was instead integrated into KinasePred for examining new compounds. This feature enables a visual identification of the specific moieties within the tested molecules that contribute most to their predicted activity, enabling a better understanding of structure–activity relationships and guiding molecular optimization by suggesting modifications to enhance activity or replace less favorable groups. Kernel SHAP was applied to compute feature importance using the kinase-family model. To coherently visualize the SHAP analysis results, we calculated atom weights using a retro-mapping approach based on binary representation [38], which was used and validated in our previous study focused on toxicity predictions for highlighting potential toxicophores in small molecules, providing a systematic framework for analyzing and interpreting molecular data. The reliability of our protocol for visualizing the SHAP analysis results and, thus, the explainability of our kinase-family ML model, were validated using randomly selected X-ray structures deposited from 2023 onward related to well-known kinase targets in complex with ligands not previously co-crystallized with other targets. This way, we ensured that the analyzed ligands were not included in ChEMBL29 and, thus, not present in the training set used for developing our kinase-family ML model.

### 3.8. Panel Inhibition Assay

The compounds selected through VS were purchased, and their inhibitory activity against various kinase proteins was evaluated using the fluorescence-based Z’-LYTE assay (Thermo Fisher Scientific, Madison, WI, USA). For this study, 20 kinases were chosen based on a protocol that prioritized targets with the highest number of associated ligands reported in the literature, indicating their relevance and scientific interest, as well as sequence diversity and clustering analysis based on sequence alignments. The inhibitory potency was measured as the percentage of inhibition in single-point experiments conducted at a concentration of 10 µM. Each measurement represented the average of two independent assays. Potential assay interference and compound fluorescence interference were thoroughly assessed across all tested conditions. Developmental interference was evaluated by comparing the test compound control wells (lacking ATP) with the 0% phosphorylation control wells (lacking the test compound). Fluorescence interference was analyzed by comparing the test compound control wells without the kinase/peptide mixture (null peptide control) to the 0% inhibition control.

### 3.9. Target-Specific Models

A total of 230 individual ML models were developed to produce target-specific predictions for 230 out of the 440 different kinase targets considered for the kinase-family model. Precisely, we selected all kinase targets presenting at least 100 dataset compounds with activity records in ChEMBL29 corresponding to IC_50_, *K*_i_, *K*_d_, or EC_50_, and with a confidence score of at least 9, retained after applying the same filtering and preprocessing steps employed to obtain the final datasets used for training, testing, and validating the kinase-family model. This way, 230 different target-specific datasets, each including at least 100 entries related to a specific kinase target, were obtained. For each target dataset, compounds with activity records corresponding to IC_50_, *K*_i_, *K*_d_, or EC_50_ values ≤ 10 μM were labeled as active, while compounds with values > 10 μM were labeled as inactive. The molecular FPs served as the input features, while the binary activity labels served as the target variable for each model. The ML model developed for each target was an RF model configured with 100 estimators, balanced class weights, and default parameters for tree depth and split criteria. A 5-fold CV scheme, where for each fold, the dataset was randomly split into training (70%) and testing (30%) subsets, was employed to ensure robust evaluation of each model. To evaluate model performance, confusion matrices were constructed for each target, and the six abovementioned statistical metrics were used. Predicted probabilities were used to classify compounds into active and inactive classes. Specifically, compounds with a predicted probability ≥ 0.75 were classified as active, while those with a probability below this threshold were classified as inactive. Model training and evaluation were implemented in Python using scikit-learn library.

## 4. Conclusions

Protein kinases are crucial for regulating cellular processes and are central to many diseases, like cancer, underscoring their importance as therapeutic targets. In this work, we developed KinasePred, a robust computational workflow for predicting the kinase activity of small molecules. By leveraging ML algorithms and explainable AI, this tool not only provides robust predictions of kinase bioactivity but also offers insights into the structural features driving ligand–target interactions. This combination of predictive power and interpretability is essential for addressing key challenges in drug discovery, such as off-target effects and selectivity optimization. The results of this study highlight the significant performance of the MLP–Morgan kinase-family model, which achieved outstanding predictive accuracy, while also providing critical interpretability through feature contribution analysis, shedding light on the molecular features driving kinase activity predictions. The best model was further validated through VS and experimental assays, which led to the successful identification of six kinase inhibitors. Building on these results, we developed specific RF models to predict the activity of small molecules against individual kinase targets. These models were validated using the compounds identified through VS. The ultimate goal of this study was the development of a comprehensive framework combining a broad-spectrum kinase-family model capable of determining whether a compound exhibits kinase activity and target-focused models designed to predict the specific kinase targets of active compounds. The broad-spectrum model is particularly useful for identifying compounds with kinase activity on multiple targets, while target-focused models can further refine predictions, providing insights into kinase selectivity and aiding the design of more selective therapeutic agents. By integrating these models, it is possible to efficiently screen compounds for kinase activity and identify their potential targets, streamlining the drug discovery process. Future efforts will focus on incorporating these models into a comprehensive platform that supports target fishing, such as polypharmacology studies and off-target identification, providing a versatile tool for drug discovery. Moreover, future studies will also be focused on further improving the predictive power and reliability of ML models thanks to more accurate molecular representations combined with more complex ML algorithms, such as the GAMIC approach, in the attempt to achieve unprecedented results in the field of in silico target fishing [39].

## Figures and Tables

**Table 1:** Performance evaluation results obtained for the developed ML models based on test set prediction.

Model	MCC	Recall	Specificity	BA	NPV	Precision
MLP–Morgan	0.96	0.99	0.98	0.98	0.99	0.99
MLP–PubChem	0.94	0.96	0.96	0.96	0.96	0.96
RF–Morgan	0.93	0.96	0.96	0.96	0.96	0.96
MLP–RDKit	0.93	0.97	0.94	0.95	0.96	0.97
RF–RDKit	0.90	0.92	0.93	0.93	0.93	0.93
RF–PubChem	0.90	0.91	0.91	0.91	0.91	0.91
GNB–Morgan	0.78	0.90	0.87	0.89	0.90	0.88
GNB–RDKit	0.59	0.83	0.75	0.79	0.82	0.77
GNB–PubChem	0.53	0.95	0.54	0.74	0.91	0.67

**Table 2:** Performance evaluation results obtained for the developed ML models based on external test set prediction.

Model	MCC	Recall	Specificity	BA	NPV	Precision
MLP–Morgan	0.88	0.90	0.98	0.94	0.90	0.97

**Table 3:** Experimental evaluation of the eight compounds against the considered kinase panel (percentage of inhibition of selected compounds ^a^).

Kinase Target	VS1	VS2	VS3	VS4	VS5	VS6	VS7	VS8
**GSK3B**	-	-	-	-	-	-	-	-
**ALK**	-	-	-	-	-	-	-	-
**RPS6KB1**	-	-	-	-	-	-	-	-
**JAK3**	-	-	-	-	-	-	-	-
**PIM2**	-	-	-	-	-	-	-	-
**mTOR**	-	-	-	-	-	-	-	-
**MAPK8**	-	-	-	-	-	63	-	-
**BRAF**	-	-	-	56	-	-	-	-
**IRAK4**	-	-	-	-	-	-	80	-
**CHEK1**	-	-	-	-	-	-	-	-
**IGF1R**	-	-	-	-	-	-	-	-
**MET**	-	-	-	-	-	-	-	-
**LCK**	-	-	-	-	-	-	53	-
**ERBB2**	-	-	-	-	-	-	-	-
**CSF1R**	-	-	-	90	54	89	89	-
**PTK2**	-	-	-	-	-	-	66	-
**NTRK1**	-	-	-	-	-	-	90	-
**SYK**	-	-	-	-	-	57	90	-
**LRKK2**	-	50	54	-	-	60	100	-
**ROCK2**	-	74	-	-	-	-	-	-

^a^ Concentration considered, 10 µM; - = percentage of inhibition < 50.

**Table 4:** Performance metrics of the two ensembles of target-specific RF models. The reported scores indicate the mean ± standard deviation across 5 CV cycles.

Models Type	Models Number	BA	MCC	Precision	Recall	NPV	Specificity
RF	230	0.83 ± 0.08	0.49 ± 0.23	0.89 ± 0.15	0.89 ± 0.08	0.55 ± 0.31	0.66 ± 0.26
RF	130	0.81 ± 0.07	0.59 ± 0.15	0.80 ± 0.16	0.85 ± 0.08	0.74 ± 0.19	0.78 ± 0.08

## Data Availability

All compounds included in the datasets used for model building and evaluation were downloaded from ChEMBL29-31 (https://www.ebi.ac.uk/chembl/, accessed on 20 January 2025) and from ZINC15 (https://zinc15.docking.org/, accessed on 20 January 2025). The calculation of molecular FPs was performed using the Python library RDKit (https://www.rdkit.org/docs/GettingStartedInPython.html, accessed on 20 January 2025). The ML models were generated and tested employing the python library scikit-learn (https://scikit-learn.org/, accessed on 20 January 2025) and TensorFlow (https://tensorflow.org, accessed on 20 January 2025). KinasePred family model is freely available at the following GitHub page: https://github.com/MMVSL/KinasePred (accessed on 19 February 2025).

## Supplementary Materials

The following supporting information can be downloaded at https://www.mdpi.com/article/10.3390/ijms26052157/s1.

## Abbreviations

The following abbreviations are used in this manuscript:

ML	machine learning
VS	virtual screening
CNN	convolutional neural networks
XAI	explainable artificial intelligence
SHAP	SHapley Additive exPlanations
LIME	Local Interpretable Model-agnostic Explanations
RF	Random Forest
GNB	Gaussian Naïve Bayes
MLP	Multi-Layer Perceptron
FPs	Fingerprints
CV	cross-validation
MCC	Matthews Correlation Coefficient
BA	Balanced Accuracy
NPV	Negative Predictive Value
ROCK2	Rho-Associated Coiled-Coil Containing Protein Kinase 2
BTK	Bruton’s tyrosine kinase
GSK-3β	Glycogen Synthase Kinase 3 Beta
TP	true positive
FP	false positive
TN	true negative
FN	false negative
