# Supplementary material for: KinasePred: A Computational Tool for Small-Molecule Kinase Target Prediction
Source: Int J Mol Sci. 2025 Feb 27;26(5):2157. doi: 10.3390/ijms26052157 (PMC11900317; doi:10.3390/ijms26052157)
Supplement: Supplementary file 1 [file ijms-26-02157-s001.zip › ijms-3479435-supplementary (1).pdf]

Supporting Information

KinasePred: A Computational Tool for Small Molecule Kinase Target Prediction

Miriana Di Stefano[1], Lisa Piazza[1], Clarissa Poles[2,5], Salvatore Galati[1,*], Carlotta Granchi[1], Antonio Giordano[3], Luca Campisi,[4] Marco Macchia[1], Giulio Poli[1,*] and Tiziano Tuccinardi[1,3]

[1]*Department of Pharmacy, University of Pisa, Pisa, Italy*

[2]*Telethon Institute of Genetics and Medicine, Naples, Italy*

[3]*Sbarro Institute for Cancer Research and Molecular Medicine, Center for Biotechnology, College of Science and Technology, Temple University, Philadelphia, PA, USA.*

[4]*Flashtox srl, Via Tosco Romagnola 136, 56025 Pontedera, PI, Italy*

[5]*Genomics and Experimental Medicine Program, Scuola Superiore Meridionale (SSM, School of Advanced Studies), Naples, Italy*

*Correspondence: salvatore.galati@unipi.it (SG) giulio.poli@unipi.it (GP)

Table of Contents

Table S1. Performance evaluation results obtained for the ML models based on CV Page S2

Table S2. Representative kinase targets used for experimental evaluations Page S3

Figure S1. Complete workflow of our study Page S4

Table S1. Performance evaluation results obtained for the ML models based on CV.

Model	MCC	BA	NPV	Precision	Recall	Specificity
MLP-Morgan	0.96 ± 0.01	0.98 ± 0.00	0.98 ± 0.00	0.97 ± 0.01	0.98 ± 0.01	0.97 ± 0.01
MLP-RDkit	0.94 ± 0.01	0.97 ± 0.00	0.97 ± 0.01	0.97 ± 0.01	0.97 ± 0.01	0.97 ± 0.01
MLP-PubChem	0.93 ± 0.00	0.97 ± 0.00	0.97 ± 0.01	0.96 ± 0.01	0.97 ± 0.01	0.96 ± 0.01
RF-Morgan	0.90 ± 0.00	0.92 ± 0.00	0.92 ± 0.00	0.92 ± 0.00	0.92 ± 0.00	0.92 ± 0.00
RF-PubChem	0.89 ± 0.00	0.93 ± 0.00	0.93 ± 0.00	0.93 ± 0.00	0.93 ± 0.00	0.92 ± 0.00
RF-RDkit	0.89 ± 0.00	0.92 ± 0.00	0.92 ± 0.00	0.92 ± 0.00	0.92 ± 0.00	0.92 ± 0.00
GNB-Morgan	0.78 ± 0.00	0.89 ± 0.00	0.90 ± 0.00	0.90 ± 0.00	0.90 ± 0.00	0.88 ± 0.00
GNB-RDkit	0.59 ± 0.01	0.79 ± 0.00	0.82 ± 0.00	0.84 ± 0.00	0.84 ± 0.00	0.75 ± 0.01
GNB-PubChem	0.55 ± 0.02	0.76 ± 0.01	0.91 ± 0.01	0.94 ± 0.01	0.94 ± 0.02	0.57 ± 0.03

Table S2. Representative kinase targets used for experimental evaluations.

Target acronym	Target name
GSK3B	Glycogen Synthase Kinase 3 Beta
ALK	Anaplastic Lymphoma Kinase
RPS6KB1	Ribosomal Protein S6 Kinase Beta-1
JAK3	Janus Kinase 3
PIM2	Proto-Oncogene Serine/Threonine-Protein Kinase Pim-2
mTOR	Mechanistic Target of Rapamycin
MAPK8	Mitogen-Activated Protein Kinase 8
BRAF	B-Raf Proto-Oncogene, Serine/Threonine Kinase
IRAK4	Interleukin-1 Receptor-Associated Kinase 4
CHEK1	Checkpoint Kinase 1
IGF1R	Insulin-Like Growth Factor 1 Receptor
MET	Hepatocyte Growth Factor Receptor, c-Met
LCK	Lymphocyte-Specific Protein Tyrosine Kinase
ERBB2	Receptor Tyrosine-Protein Kinase erbB-2, HER2
CSF1R	Colony Stimulating Factor 1 Receptor
PTK2	Protein Tyrosine Kinase 2
NTRK1	Neurotrophic Receptor Tyrosine Kinase 1
SYK	Spleen Tyrosine Kinase
LRRK2	Leucine-Rich Repeat Serine/Threonine-Protein Kinase 2
ROCK2	Rho-Associated Coiled-Coil Containing Protein Kinase 2

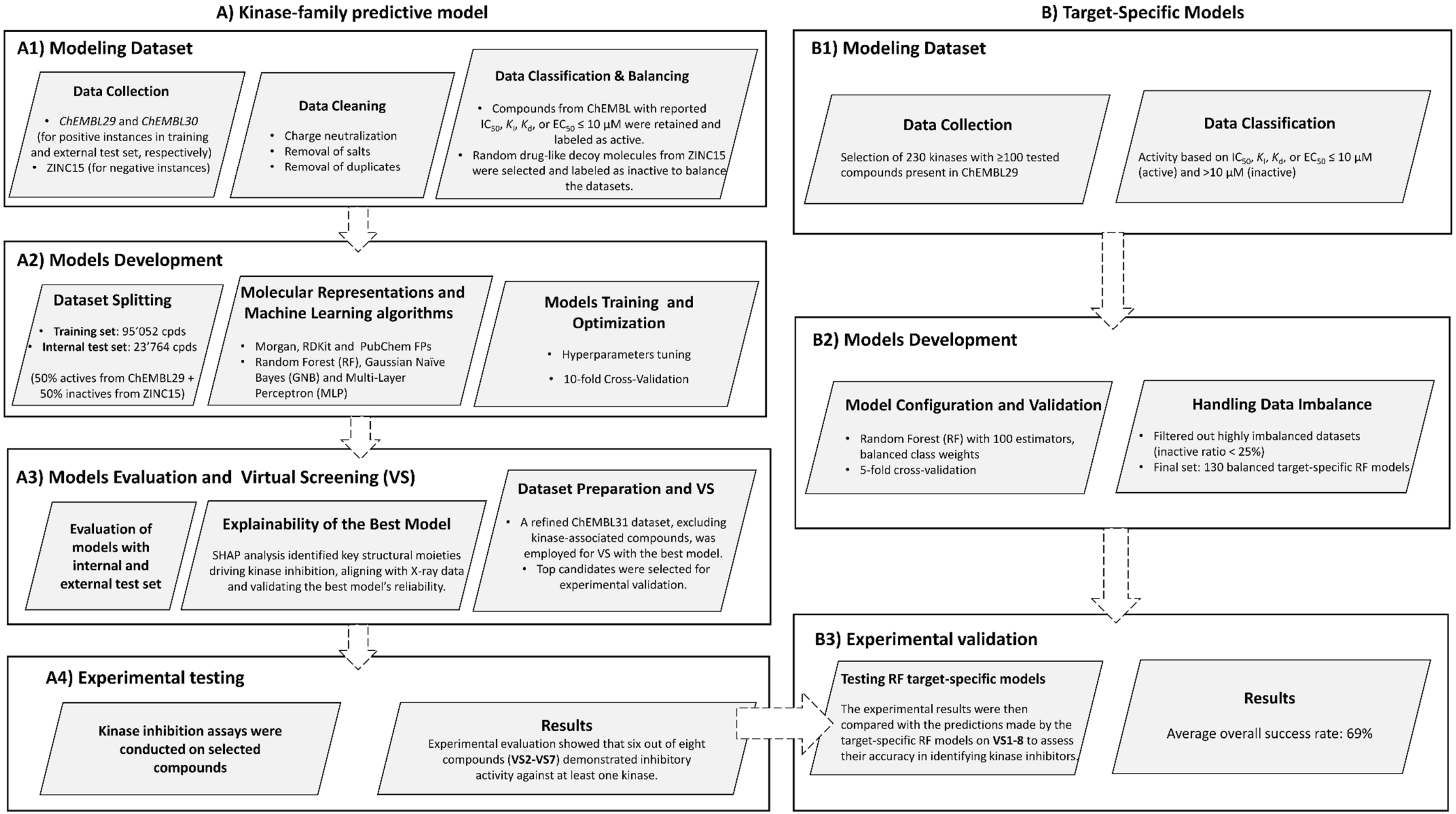

Figure S1. Complete workflow of our study, including the step-by-step procedures followed for developing the kinase-family predictive model and performing the virtual screening, as well as for generating the target-specific models and validated them based on the experimental results.
